# Engineering complex tissue-like microgel arrays for evaluating stem cell differentiation

**DOI:** 10.1038/srep30445

**Published:** 2016-07-28

**Authors:** Enrico Guermani, Hossein Shaki, Soumyaranjan Mohanty, Mehdi Mehrali, Ayyoob Arpanaei, Akhilesh K. Gaharwar, Alireza Dolatshahi-Pirouz

**Affiliations:** 1 Department of Mechanical Engineering, Politecnico di Torino, Corso Duca degli Abruzzi 24, 10129 Torino, Italy; 2 Technical University of Denmark, DTU Nanotech, Center of Nanomedicine and Theranostics, Kongens Lyngby, 2800 Kgs. Lyngby, Region Hovedstaden, Denmark; 3 Department of Biotechnology, Faculty of Chemical Engineering, Tarbiat Modares University, Teheran, Iran; 4 Technical University of Denmark, DTU Nanotech, Kongens Lyngby, 2800 Kgs. Lyngby, Region Hovedstaden, Denmark; 5 Department of Industrial and Environmental Biotechnology, National Institute of Genetic Engineering and Biotechnology, Tehran, Iran; 6 Department of Biomedical Engineering, Texas A&M University, College Station, TX 77843, USA; 7 Department of Materials Science and Engineering, Texas A&M University, College Station, TX 77843, USA

## Abstract

Development of tissue engineering scaffolds with native-like biology and microarchitectures is a prerequisite for stem cell mediated generation of off-the-shelf-tissues. So far, the field of tissue engineering has not full-filled its grand potential of engineering such combinatorial scaffolds for engineering functional tissues. This is primarily due to the many challenges associated with finding the right microarchitectures and ECM compositions for optimal tissue regeneration. Here, we have developed a new microgel array to address this grand challenge through robotic printing of complex stem cell-laden microgel arrays. The developed microgel array platform consisted of various microgel environments that where composed of native-like cellular microarchitectures resembling vascularized and bone marrow tissue architectures. The feasibility of our array system was demonstrated through localized cell spreading and osteogenic differentiation of human mesenchymal stem cells (hMSCs) into complex tissue-like structures. In summary, we have developed a tissue-like microgel array for evaluating stem cell differentiation within complex and heterogeneous cell microenvironments. We anticipate that the developed platform will be used for high-throughput identification of combinatorial and native-like scaffolds for tissue engineering of functional organs.

The ability to create functional tissues structures from biomaterials, cells and biochemical signaling molecules would enable significant advances in tissue engineering, organ repair and drug screening^1^,^2^,^3^. To engineer artificial tissue constructs it is essential to preciously sculpture various building blocks including cells, extracellular matrix (ECM) proteins and materials into native-like tissue architectures^4^,^5^,^6^,^7^,^8^,^9^. These components play a combinatorial role in maintaining, supporting and sustaining tissue functions in the human body. However, a range of challenges are needed to be addressed before organs with tissue-like architectures can be fabricated to control, maintain and trigger cell phenotype^10^,^11^,^12^,^13^. Recently, 3D printing of hydrogel-based bioinks loaded with cellular components into tissue-like architectures has been investigated to engineer complex tissue structures^14^,^15^,^16^,^17^. However our inability to print multiple cell types within a tissue construct and understanding the combinatorial effect of cells, ECM and soluble factors have prevented the 3D bioprinting technology to address the key challenges in regenerative medicine^18^. One of the emerging strategies to address this unmet need is to engineer cell-laden microgel arrays for combinatorial and cost-effective identification of the important physical, biological and geometrical factors for engineering functional tissues^19^,^20^,^21^,^22^,^23^.

Currently 3D bioprinting has been used to microprint cell-laden microgel arrays for high-throughput screening of various biological factors to identify key factors responsible for stem cell differentiation^21^,^22^,^24^,^25^,^26^. While elegant, these bioprinting platforms are devised for screening simple microenvironments composed of homogenous cell-laden matrixes or growth factors. However, to date, none of these microprinting technologies have been used to produce structurally complex cell-laden microgel arrays. By using bioprinting technology, it is possible to print cell-laden hydrogel bioinks with tissue-like architectures. It is expected that through this technology it is possible to evaluate a range of biomaterials with different physical, biological, geometrical and cellular heterogeneities. Recently, a range of studies has demonstrated the application of layer-by-layer printing techniques for engineering complex tissue constructs^27^,^28^,^29^. However, none of these microprinting technologies have been shown to generate structurally complex cell-laden microgel arrays.

For successful microprinting of hydrogel bioinks into structurally complex microgel arrays three general conditions must be meet: (a) the printed microgels must retain a 3D shape after deposition, (b) a homogenous 3D cell distribution is required inside microgels and (c) a high cell viability (>80%) must be sustained over a period of at least 7 days. Here, we report a new microprinting approach to print complex cellular structures that mimick native tissue architectures using photocrosslinkable bioinks for facile cell encapsulation. It is our expectation that by generating arrays of complex tissue structure it is possible to understand effect of structural, cellular and microenvironmental heterogeneity on human mesenchymal stem cells (hMSCs) differentiation. To our knowledge this is the first approach to demonstrate the application of microprinting technology for the manufacture of microgel arrays with tissue-like architectures. It is expected that this technology can be used to understand cellular behavior within multi-component tissue constructs in a high-throughput approach.

## Results and Discussion

3D printing of tissue-like geometries requires an optimal printing strategy and suitable bioinks (i.e. hydrogels) to ensure high cell survival, good architecture fidelity, homogenous cell distribution and long-term platform stability. We propose to use gelatin methacrylate (GelMA) and thiolated hyaluronic acid (HA-SH) as model systems, to print stem cells into complex structures. We used a robotic microspotter for automated deposition of cell-laden GelMA and HA-SH in a temperature and humidity controlled environment to preserve high cell viability.

**Figure 1:**
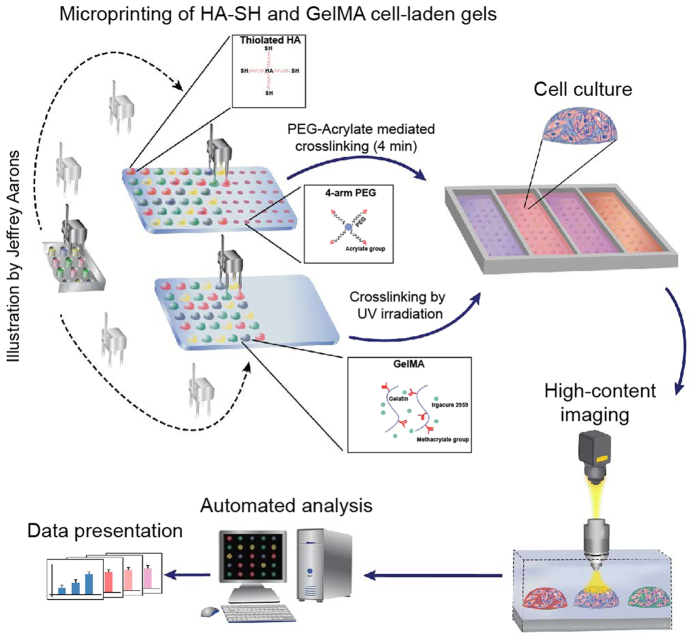
Fabrication of hydrogel microgel arrays. Schematic of the fabrication process showing the deposition of cell-laden pre-polymers (HA or GelMA) on functionalized glass slides (Epoxy or TMSPMA). The pre-polymer solution was crosslinking either though thiol-ene reaction (HA-PEG)) or through a UV-irradiation step (GelMA). UV-exposure time was 15 sec and two different HA crosslinker concentrations was used (1% and 0.5%) (Illustration made by Jeffrey Aarons).

To bioprint microgel constructs, prepolymer solutions consisting of hMSCs were deposited on functionalized glass slides (25 mm × 75 mm) (Fig. 1). The bioinks were deposited on 3-trimethoxysilyl) propyl methacrylate (TMSPMA) and epoxy functionalized substrates to provide stability of printed microgels up to 10 days of culture. The cell-laden prepolymer GelMA microdrops were crosslinked through free radical polymerization mediated by UV irradiation in presence of photoinitiator (Irgacure-2959). Cell-laden prepolymer HA-SH microdrops were crosslinked by depositing HA-SH prepolymer solutions onto microspots consisting of four-arm PEG-acrylate. The acrylate groups on PEG initiated crosslinking by linking to thiol groups present on HA-SH polymers (Fig. 1). Microprinting of both cell-laden GelMA and HA-SH resulted in 3D microdrops, which retained their 3D profile until gelation occurred.

To evaluate the modularity of our system, HA-SH microgel with different stiffness was fabricated by controlling the PEG-acrylate concentration. Two PEG-acrylate concentrations (0.5% and 1%) were used with HA-SH resulting in compressive modulus at 5 kPa and 10 kPa, respectively. The four-pin microprinter enabled bioprinting of microgel (>600 spots) on a glass cover slip in a few minutes. The bioprinted microgels were stable for more than 10 days in physiological conditions and no significant detachment of microgels from the glass surface was observed.

**Figure 2:**
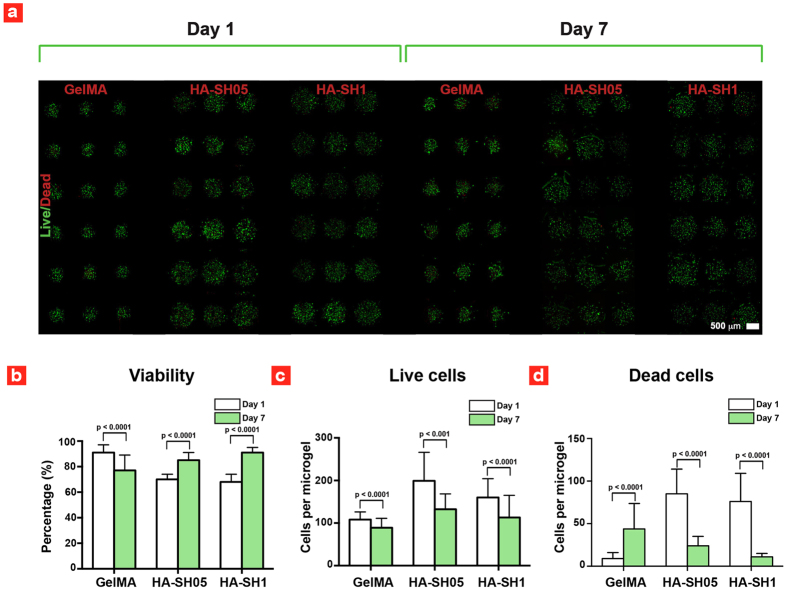
Cell viability inside printed microgels. (**a**) Fluorescence images of dead (**red**) and live (**green**) hMSCs. (**b**) hMSCs viability, (**c**) number of live hMSCs and (**d**) number of dead hMSCs. GelMA viability data are taken with permission from ref. 25.

As previously reported^25^ hMSCs encapsulated within GelMA microgels showed a high cellular viability (91 ± 6%) at day 1 and a slightly lower viability (78 ± 6%) at day 7 (Fig. 2a). Whereas the cell survival rate within HA-SH microgels was markedly different with a low cellular viability (69 ± 2%) at day 1 and a higher cell viability (85–91%) at day 7 (Fig. 2b). To investigate the effect of polymer on cell viability, 3D live/dead imaging of entrapped cells were performed (Fig. 2b). The number of live cells in HA-SH and GelMA microgels was similar on day 1 and day 7, while the number of dead cells in HA-SH microgels was drastically reduced between day 1 and day 7. This suggests that non-adherent apoptotic hMSCs are not retained within HA-SH microgels and result in an increase in the percentage of live cells over the course of 7 days.

**Figure 3:**
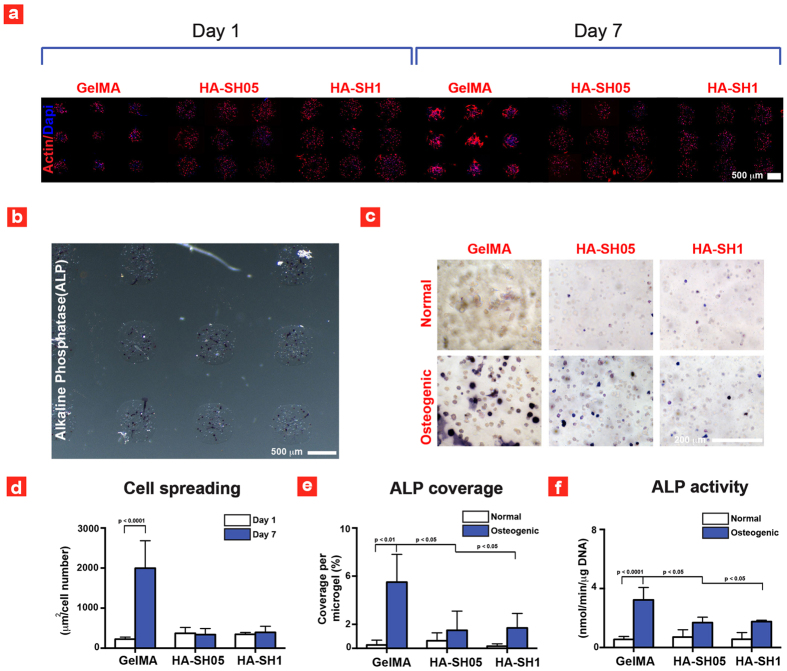
Cell spreading and differentiation within the deposited microgels. (**a**) Fluorescence images of hMSCs within the printed microgels. (**b**) Optical image of ALP stained gel microgel array and (**c**) optical images showing the ALP expression inside different microgels. (**d**) Quantified cell spreading data after 1 and 7 days of culture. (**e**) ALP coverage of the different constructs at day 7 and (**f**) Analyzed ALP activity after 7 days of culture. GelMA ALP data in (**f**) is taken with permission from ref. 25.

Another condition that must be meet for successful bioprinting is that the printed hydrogel bioinks enable cell spreading and can induce lineage-specific differentiation. To test this requirement cost-effectively, we explored the osteogenic differentiation and cell-spreading capability of microgel array encapsulated hMSCs. For example, high cell spreading has shown to promote osteogenesis, while low cell-matrix interaction result in round shaped morphology that facilitates chondrogenesis The results showed that hMSCs within GelMA reached near confluency after 7 days of culture, while non-existent cell-spreading of encapsulated hMSCs was observed within HA-SH microgels (Fig. 3a). Moreover, on day 7, cells seeded on GelMA indicate presence of stretch actin filaments and thus highlight strong cell-matrix interactions. Whereas no stretch actin filaments were observed in HA-based microgels indicating low cell-matrix interactions.

The effect of GelMA (promote cell adhesion) and HA-SH (suppress cell spreading) was investigated on osteogenic differentiation of hMSCs. The osteogenic differentiation of encapsulated hMSCs was determined using alkaline phosphatase (ALP) staining and activity. ALP is an early marker for osteogenesis of hMSCs. As previously reported^25^ the results indicate that cells encapsulated within GelMA hydrogels showed significantly higher ALP staining in osteogenic media compared to normal media (Fig. 3b,c). The quantification of cell spreading and ALP coverage demonstrated that GelMA led to a significantly higher cell spreading and ALP expression compared to HA-SH. This indicates that cell adhesion is necessary to facilitate differentiation of hMSCs towards osteogenic lineages in accordance with previous findings^30^. To further quantify the ALP results, the ALP activity of encapsulated hMSCs was determined using macrogels (Fig. 3f). The ALP activity observed in macroscale setting (Fig. 3(f)) confirmed that cells encapsulated within GelMA microgels showed significantly higher osteogenic differentiation compared to cells seeded in HA microgels. From high-throughput screening, we conclude that GelMA is the preferred microgel system for bioprinting compared to HA, since GelMA ensures higher cell viability (>80%), enhances cell adhesion and spreading and promotes osteogenic differentiation of hMSCs in accordance with previous findings. Based on these findings we designed a new set of experiments with the goal to print tissue-like microgel arrays.

**Figure 4:**
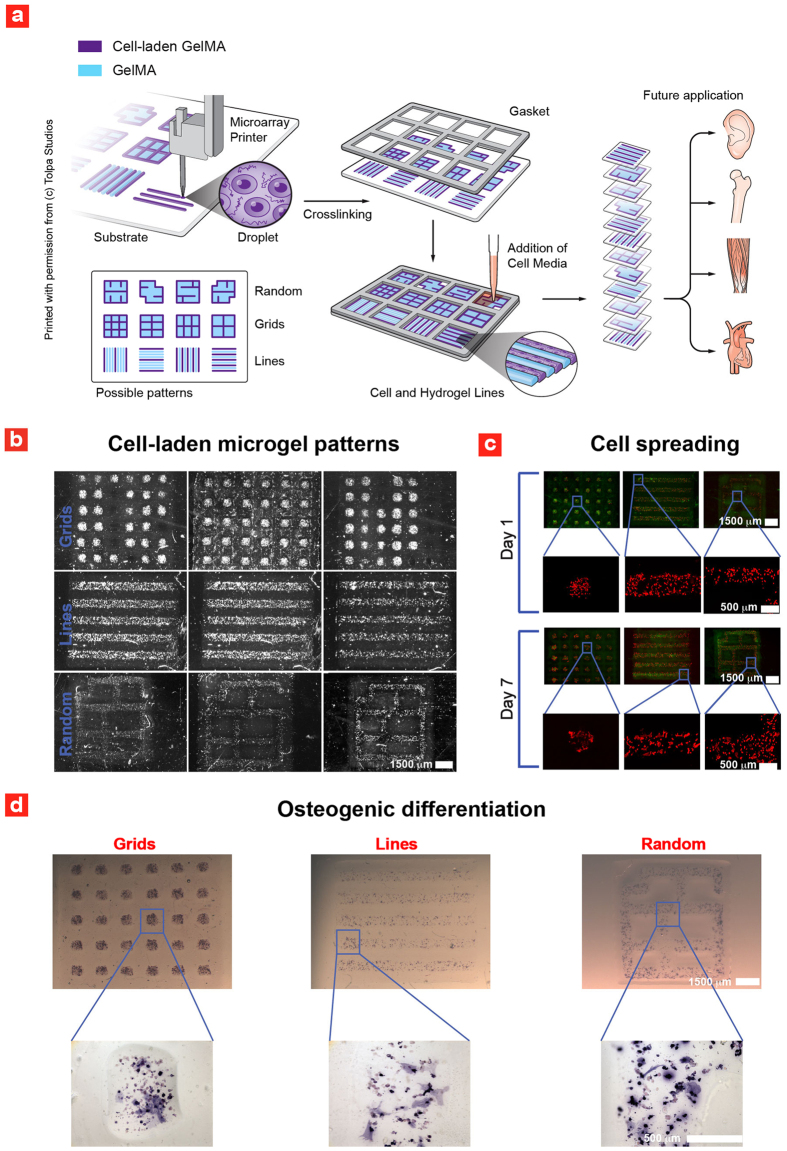
Microdrop printing of cell-laden hydrogels into native-like tissue architectures. (**a**) Schematic of the fabrication process and future applications of the developed platform (Illustration made by Tolpa Studios). (**b**) Various printed cell-laden microgel patterns. (**c**) hMSCs spreading within the patterned microgels (Green is obtained by filtering the brightfield channel with imageJ to remove noise-peaks). (**d**) Spatially controlled differentiation of hMSCs inside the patterned microgels.

Another important requirement in tissue engineering is localized differentiation of stem cells into random and well-ordered tissue architectures. Well-ordered tissue architectures are found throughout the human body, while random tissue architectures are seen in the bone marrow, which consist of a porous structure coined “spongy bone”. For proof-of-concept, we used GelMA to print cell-laden lumen architectures, grid-like architectures and random architectures that bear resemblance to spongy bone. Microscopic cell-laden gels were printed in a layer-by-layer fashion from small microdroplets to generate the complex architectures (Fig. 4a). High-fidelity was achieved by mixing the GelMA prepolymer solution with 15% glycerol before printing. Subsequently, the printed microgel structures were exposed to UV irradiation to develop the tissue-like microgel array platform. Following the 3D printing process, the miniaturized platform was cultured in a gasket chamber, that enabled different culturing conditions for each printed microgel architecture (Fig. 4a). Such combinatorial culturing conditions are a necessity to determine the right media-formulations in designing complex and heterogeneous tissues. After 1 day of culture in gasket chambers optical imaging of the platform was performed. The acquired images revealed a localized distribution of hMSCs into 3D lumen-like, grid-like and random architectures (Fig. 4b). These trends were further validated using fluorescence imaging to determine cell cytoskeleton, which indicates homogenous distribution of hMSCs within the microgels (Fig. 4c). After 7 days of culture, cells with well-defined cytoskeleton was observed, a prerequisite for the design of complex tissue architectures.

To investigate the lineage-specific differentiation of encapsulated hMSCs into osteogenic cells, ALP staining of the generated tissue-like microgel array was performed (Fig. 4d). These results clearly demonstrated that hMSCs remained localized within the bioprinted structures and differentiate into osteogenic lineage after 7 days of culture. This trend was observed yet again from high-magnification images of the cell-laden microgel architectures. These results confirm that microdrop printing of cell-laden GelMA can be used to generate complex and heterogeneous cellular microconstructs. This will also provide a roadmap for the development of tissue-like microgel arrays through a facile microprinting method.

HMSCs remained localized inside the bioprinted tissue-like geometries for seven days with high cell viability and were able to differentiate into osteogenic lineage. However, it was difficult to maintain the stability of the bioprinted microgel architectures. We believe this was mostly due to the degradation of hydrogel network by collagenase secreted by hMSCs, and/or cell migration and spreading. The developed microgel platform could be improved by combining GelMA with other natural/synthetic polymer that can withstand collagenase degradation such as hyaluronic acid, alginate and PEG.

## Conclusion

In summary we have demonstrated a novel microprinting approach of cell-laden bioinks into 3D tissue-like architectures through a facile microprinting strategy. This strategy can be used to bioprint tissue-like microgel arrays with complex multicomponent systems. We anticipate that screening of cell fate within these microarrayed architectures will open up new avenues for fabrication of heterogeneous tissues. In future, this high-throughput platform can be used to examine stem cell fate within such heterogeneous microconstructs, enabling a cost-effective development of new tissue engineering strategies. While several cell-printing studies have demonstrated successful printing of various living tissue architectures, our results is the first to demonstrate 3D printing of cell-laden bioinks into tissue-like arrays.

## Methods

### Cell culture

Human mesenchymal stem cells (hMSCs) were acquired from Lonza (Lonza, MD) at passage 2. The hMSCs were cultured in a T-75 flask in a commercially available stem cell growth media (MSCSGM) (Lonza, MD) specific for mesenchymal stem cells and cells were grown and passaged according to previous work^31^,^32^. Osteogenic experiments were carried out by stimulating hMSCs into the osteogenic lineage through cell culturing in an osteogenic media (Lonza, MD) that was changed twice a week. The osteogenic cell media were made from MSCSGM media and contained L-Glutamine, Ascorbate, Pen/Strep, Dexamethasone and β-Glycerophosphate.

### Hydrogel synthesis

GelMA was generated according to previous work^33^,^34^. In this protocol 10 g of gelatin (Sigma) was mixed in 100 ml PBS and methacrylation was generated through addition of 8% (v/v) methacrylic anhydride to the pre-polymer solution for 3 hours at 50° C. The finalized methacrylated pre-polymer solutions were purified through a 12–14 kDA dialysis. The purification process lasted up to 7 days. After the purification process, the GelMA solution was frozen at −80° C and freeze-dried for 4 days. Crosslinked hydrogels were obtained by mixing 5% (w/v) GelMA with 0.5% (w/v) Irgacure 2959 photoinitiator ((2-hydroxy-1-(4-(hydroxyethoxy)phenyl)-2-methyl-1-propanone) and exposing the pre-polymer solution to UV-light for 15 seconds (320–500 nm, 800 mW power, 10 cm height). Previous studies have shown that Irgacure 2959 do not trigger any severe biological responses as such low doses^35^. Thiolated hyaluronic acid (HA-SH) was purchased from a commercial source (Biotime) and gelation was initiated by addition of a four-arm poly(ethylene glycol) acrylate (PEGTA) (Creative PEGWorks) to the HA-SH solution through a michael-addition process.

### Microprinting

The microprinting was either performed on glass-slides functionalized with 3-(trimethoxysilyl)propyl methacrylate (TMSPMA)(Arrayit) or epoxy functionalized glass-slides (Arrayit) using a SpotBot 3 microprinter (Arrayit). GelMA was deposited on TMSPMA, while HA-SH was deposited on epoxy glass-slides in a temperature and humidity controlled environment (22° C and 90%, respectively). After the deposition process the microgel arrays were cultured inside sealed multiwall culture chambers (GraceBio) containing MSCGM media. Replacing MSCGM media with osteogenic media after 24 hours induced osteogenic differentiation. After 7 days of culture images of the microgel arrays were obtained using a Zeiss Axio Observer Z1 and image analysis was conducted in a time-efficient manner using National Institute of Health (NIH) ImageJ software (v. 1.4).

### Confocal imaging

A Leica TCS SP5 microscope containing a 405 nm diode and a white light laser tuned to 495 nm (Leica Microsystems) was used for confocal imaging. Confocal images were with Imaris v7.6.1 software (Bitplane).

### Viability analysis

Cell survival rates were quantified through a commercially available Live/Dead kit (Invitrogen). Briefly the cell-laden microgel chips were exposed to a solution consisting of calcein (0.5 μl/ml) and ethidium homodimer (ETD) (2 μl/ml). Following the viability staining the slides were rinsed three times and imaged (Zeiss Axio Observer). The data was auto-analysed using a build-in script in the ImageJ v.1.4. software, which uses particle segmentation to pick-out the live and dead cells respectively.

### Osteogenic differentiation

Microgel chips were fixed in 4%(v/v) paraformaldehyde (Electron Microscopy Sciences) for 20 min and osteogenic lineage of the cells was invistigated using Alkaline Phosphatase (ALP) as an early osteogenic differentiation marker. A standard solution consisting of BCIP/NBT (MP biomedical) was employed for evaluating ALP expression. The BCIP/NBT solution was added to the microgel chips and incubated for 90 min. Finally the microgel chips were rinsed three times in PBS before imaging. ALP activity was measured by digesting gels with 100 μg/ml dispase II (Sigma). The digested gels were added to a 96-well plate and the ALP activity was analyzed with standardized colorimetric Assay kit (Abcam) in which the optical density (O.D.) of the different samples were evaluated at 405 nm using a SpectraMax M5 spectrometer (Molecular devices) from know standard curves. ALP activity was normalized with the DNA content of the respective samples through a commercially available Quant-iT PicoGreen kit (Invitrogen) by following the manufactures protocol.

### Statistics

The level of significance among the experimental groups was performed through a two-tailed t-test, when the data was normally distributed, or a non-parametric Mann-Whitney test. Averaged were considered to be significantly different when p-values were less than 0.05. All the data presented herein is presented as average ± standard deviation (SD) and the statistical analysis was carried out with JMP software (vs. 10).

## Additional Information

**How to cite this article**: Guermani, E. *et al*. Engineering complex tissue-like microgel arrays for evaluating stem cell differentiation. *Sci. Rep.* **6**, 30445; doi: 10.1038/srep30445 (2016).
